# 3D vs. 4K Display System - Influence of “State-of-the-art”-Display Technique On Surgical Performance (IDOSP-Study) in minimally invasive surgery: protocol for a randomized cross-over trial

**DOI:** 10.1186/s13063-019-3330-7

**Published:** 2019-05-28

**Authors:** Roger Wahba, Rabi Raj Datta, Andrea Hedergott, Jana Bußhoff, Thomas Bruns, Robert Kleinert, Georg Dieplinger, Hans Fuchs, Caroline Giezelt, Desdemona Möller, Martin Hellmich, Christiane J. Bruns, Dirk L. Stippel

**Affiliations:** 1 Department of General, Visceral and Cancer Surgery, University Hospital of Cologne, University of Cologne, Kerpener Straße 62, 50937 Cologne, Germany; 2 Department of Ophthalmology, University Hospital of Cologne, University of Cologne, Cologne, Germany; 3 Faculty of Management, Economics and Social Sciences, Department of Business Administration and Health Care Management, University of Cologne, Cologne, Germany; 4 Institute of Medical Statistics and Computational Biology, Faculty of Medicine and University Hospital of Cologne, University of Cologne, Cologne, Germany

**Keywords:** Minimally invasive surgery, Laparoscopic, 3D, 4K, Surgical performance, Learning curve, Surgical training

## Abstract

**Background:**

Three-dimensional (3D) stereoscopic vision is crucial to perform any kind of manual task. The reduction from real life 3D to virtual two-dimensional (2D) sight is a major challenge in minimally invasive surgery (MIS). A 3D display technique has been shown to reduce operation time and mistakes and to improve the learning curve. Therefore, the use of a3D display technique seems to optimize surgical performance for novice and experienced surgeons. Inspired by consumer electronics, a 4K display technique was recently introduced to MIS. Due to its high resolution and zoom effect, surgeons should benefit from it. The aim of this study is to evaluate if “state-of-the-art” 3D- vs. 4K-display techniques could influence surgical performance.

**Methods:**

A randomized, cross-over, single-institution, single-blinded trial is designed. It compares the primary outcome parameter “surgical performance”, represented by “performance time ”and “number of mistakes”, using a passive polarizing 3D and a 4K display system (two arms) to perform different tasks in a minimally invasive/laparoscopic training parkour. Secondary outcome parameters are the mental stress load (National Aeronautics and Space Administration (NASA) Task Load Index) and the learning curve. Unexperienced novices (medical students), non-board-certified, and board-certified abdominal surgeons participate in the trial (i.e., level of experience, 3 strata). The parkour consists of seven tasks (for novices, five tasks), which will be repeated three times. The 1st run of the parkour will be performed with the randomized display system, the 2nd run with the other one. After each run, the mental stress load is measured. After completion of the parkour, all participants are evaluated by an ophthalmologist for visual acuity and stereoscopic vision with five tests. Assuming a correlation of 0.5 between measurements per subject, a sample size of 36 per stratum is required to detect a standardized effect of 0.5 (including an additional 5% for a non-parametric approach) with a power of 80% at a two-sided type I error of 5%. Thus, altogether 108 subjects need to be enrolled.

**Discussion:**

Complex surgical procedures are performed in a minimally invasive/laparoscopic technique. This study should provide some evidence to decide which display technique a surgeon could choose to optimize his performance.

**Trial registration:**

ClinicalTrials.gov, NCT03445429. Registered on 7 February 2018.

**Electronic supplementary material:**

The online version of this article (10.1186/s13063-019-3330-7) contains supplementary material, which is available to authorized users.

## Background

Laparoscopic and minimally invasive operation techniques/surgery (MIS) have become the standard in basic (e.g. cholecystectomy [1]) as well as in complex surgical procedures (e.g. living donor nephrectomy [2]). In general, the learning curve for MIS is prolonged compared to open surgery [3] and even longer for complex operations [4]. One challenge is the reduction from real life three-dimensional (3D) stereoscopic vision to virtual two-dimensional (2D) sight. 3D vision is very important to perform any kind of manual task [5]. Therefore, optimizing the visualization of the operative field is required, especially in MIS. A 2D full-high-definition technique was one step used to improve vision. The passive polarizing 3D display technique reintroduces natural stereoscopic view and orientation to MIS. It leads to shorter operation times and seems to optimize surgical performance compared to standard 2D imaging in basic procedures [6, 7]. Novices as well as experienced surgeons seem to benefit from the 3D passive polarizing technique [8]. The learning curve and performance, especially in complex surgical procedures, e.g. vascular preparation during retroperitoneoscopic donor nephrectomy, could be optimized and simplified [9]. The recent European Association for Endoscopic Surgery (EAES) consensus statement recommended the use of 3D vision to reduce operative time [7]. As a disadvantage of the technique, the surgeon must wear glasses and the equipment is expensive. Furthermore, a relevant percentage of the population has deficits in binocular and stereoscopic vision, which could induce dizziness and nausea when using the passive polarizing 3D video technique [5]. This could result in a deterioratingsurgical performance. Inspired by consumer electronics, the 4K-display technique has reached medicine. It creates a high resolution image with 4098 × 2160 pixels on a large-scale 55″ monitor (140 cm), resulting in an up to 30 times zoom. Due to these features, it should also optimize surgical performance in MIS and could be an alternative to the passive polarizing 3D display technique. Data comparing these techniques are scarce. Therefore, both techniques are compared in this randomized cross-over setting. The aim of this study is to evaluate if “state-of-the-art” display techniques could influence surgical performance, represented by the outcome parameters “performance time ”and “number of mistakes” in different tasks of a minimally invasive/laparoscopic training parkour.

## Methods/design

**Fig. 1:**
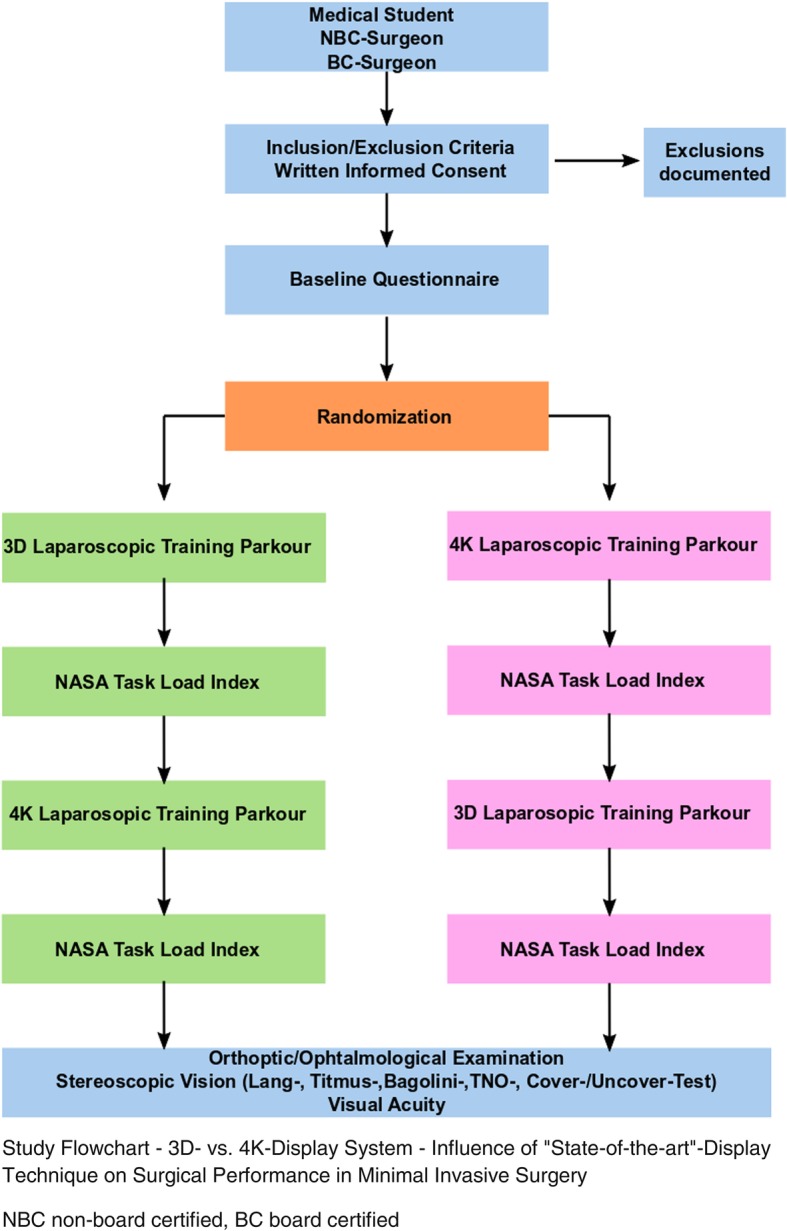
Study Flowchart - 3D vs. 4K Display System - Influence of “State-of-the-art”-Display Technique on Surgical Performance in Minimally Invasive Surgery. *NBC* non-board certified, *BC* board certified

A randomized, cross-over, single-blinded trial is designed. It compares the surgical performance in a laparoscopic/MIS training parkour using a passive polarizing 3D and 4K display system. One should test whether the tasks of the training parkour can be performed faster and/or with fewer mistakes using one of the display systems. The influence of the display technique on the learning curve will also be evaluated. The trial is performed at a single institution (Department of General, Visceral and Cancer Surgery, University Hospital of Cologne). Subjects of the study will be surgeons from the University Hospital of Cologne as well as from primary and secondary hospitals/community clinics in Cologne. After written informed consent (obtained by RW, RD, JB, or TB), subjects will be randomized to start the training parkour with the 3D or the 4K system. After completion of the parkour with the first display system, the task load is evaluated by the National Aeronautics and Space Administration Task Load Index (NASA-TLX). After that, the parkour is performed with the other display system (vice versa setting), followed again by NASA-TLX. Three different groups (i.e., strata) of subjects participate in the trial: unexperienced novices to MIS (medical students), non-board-certified abdominal surgeons in training with some experience in MIS, and board-certified abdominal surgeons with a high level of experience in MIS. The parkour consists of 7 tasks (novices 5 tasks), which will be repeated three times. After completion of the parkour, all participants are examined by an ophthalmologist for stereoscopic vision and exclusion of manifest strabismus with five qualitative and semi-quantitative tests: Lang (I and II)-, Titmus-, Bagolini striated glasses test (near and far distance test), TNO stereo tests (near distance) and cover−/uncover test (near and far distance). Further, monocular visual acuity is tested (far distance) and the anterior segment and central fundus are screened for relevant anomalies. Figure 1 shows the study flowchart. According to the Standard Protocol Items: Recommendations for Interventional Trials (SPIRIT) 2013 guidelines, a trial schedule (Table 1) and a trial checklist (Additional file 1) are part of the protocol [10, 11].

**Table 1:** Trial Schedule – 3D vs. 4K Display System - Influence of “State-of-the-art”-Display Technique on Surgical Performance

Schedule	Enrollment	Laparoscopic Parkour 1st Display system	Laparoscopic Parkour 2nd Display system	Orthoptic/Ophthalmologic Examination
Assessment No.	1	2	3	4
Time	−28 until Day 1	Day 1	Day 1	Day 2 until 21
In−/exclusion criteria	x			
Informed consent	x			
Registration	x			
Questionnaire Baseline Characteristics	x			
Randomization	x			
Surgical Performance		X	X	
-time				
-mistakes				
NASA-TLX		X	X	
Orthoptic/ophthalmologic examination				x

Each x signifies one study specific event, which is documented for later evaluation. The trial schedule is created according the SPIRIT guideline and figure [10, 11]

### Outcome measures and data collection

The primary outcome measure is the surgical performance measured by the items “time in seconds” and “number of mistakes”. Both items are measured for each task separately and for all tasks together. The mistakes are defined for every task as any deviation from perfect performance (general and special mistakes). Secondary outcome parameters are the scores of the NASA-TLX and the learning curves. Learning curves will be described as performance (time, errors) over repetitions with an added standard CUSUM analysis [12]. Moreover, performance indicators are investigated for possible interaction of replication, technique, and sequence (3D after 4K or vice versa).

Baseline characteristics are acquired by a questionnaire. The MIS tasks are recorded as standard 2D videos. NASA-TLX is performed as a pen-and-paper version. An ophthalmological examination is performed and documented on a separate case report form (CRF). When the data for one subject are complete, they will be transferred to the data trustee, who pseudonymized the data and videos. The videos will be sent back to the investigators for evaluation of the primary outcome measure. Each video will be assessed by two evaluation-trained investigators. Inter-rater reliability will be evaluated by contingency table analysis (kappa statistics or intraclass correlation). Large differences, i.e., larger than 1.96*standard deviations, will be reevaluated by two additional raters and described qualitatively. This will be documented on a CRF and retransferred to the data trustee, who will reunite it all. Then the pseudonymized data are sent to the investigators and statistician for final evaluation. Table 1 shows the type and time of data collection. At the time of publishing the study protocol, the trial is still recruiting.

### Inclusion criteria

Subjects fulfilling the following inclusion criteria may be enrolled in the study:

1. Medical student, surgeons in training, board-certified surgeons
2. Those who have given written informed consent
3. Those aged > 18 years

### Exclusion criteria

The following criteria will exclude subjects from the study:

1. Medical students with any experience in laparoscopic surgery
2. Experience in the laparoscopic training parkour (all subjects)
3. Non-correctable vision disorders
4. Known impaired stereoscopic vision
5. Manual skill disorders

### Randomization and blinding

Randomization of subjects to sequences is based on permuted blocks and stratified by level of experience. This will be performed by the data trustee. After that subjects will perform the laparoscopic parkour and the NASA-TLX and be examined by the ophthalmologist. The performance of the laparoscopic parkour will be video documented as standard 2D videos. After completion of the study examination, all data including the videos will be collected by the data trustee and pseudonymized and stored on a secured data base with routine backup. To guarantee blinding, only pseudonymized data are sent back to the investigators for evaluation of the final study data. Therefore, the evaluating investigators are not able to find out whether the 3D or 4K display system was used during the laparoscopic training parkour.

### Data management

Data evaluation and entry to the study data base will be double checked and performed by two investigators. Final access to the data base is given to the sponsor, responsible party, and authors of the protocol. It will not be provided to any third party.

### Interim analysis and stopping guidelines

There is no interim analysis planned. There are no stopping guidelines due to the fact that the trial does not evaluate an US –Food and Drug Administration (FDA)-regulated drug product or a US FDA-regulated device product.

### Sample size

Assuming a correlation of 0.5 between measurements per subject, a sample size of 36 per stratum is required to detect a standardized effect of 0.5 (including an additional 5% for a non-parametric approach) with a power of 80% at a two-sided type I error of 5%. This is a cautious estimate since a considerably larger effect size of 1.0 was reported by Smith et al. [8] for the improvement in the median time and for completion of the entire protocol, albeit in a parallel-group setting. Similarly, for the median number of errors, an effect size of 1.95 was observed. Also, preliminary cases supported the sample size calculation. Thus, altogether 108 subjects need to be enrolled [8]. Subjects who drop out of the study may be replaced.

### Statistical analyses

Quantitative variables are summarized by mean ± standard deviation and percentiles (0, 25, 50, 75, and 100), qualitative variables by count and percentage. Outcome measures are evaluated by modeling; specifically (generalized) linear mixed models for repeated measures (MMRM) with main effects modality, stratum, and period (type III SS, REML, unstructured covariance matrix). Estimated marginal means and contrasts are derived. Interaction effects, particularly stratum*modality, are explored. Two-sided *p* values < 0.05 are interpreted to indicate statistical significance. Missing data will substituted by multiple imputations. Subgroup analysis will be performed according to the above mentioned strategies.

### Trial organization

The IDOSP trial is an investigator-initiated trial without external funding. The trial is sponsored by the University Hospital of Cologne. The Department of General, Visceral and Cancer Surgery is responsible for the coordination of the trial.

#### Ethics

Ethics Committee approval was obtained before the study (Ethikkommission der Medizinischen Fakultät der Universität zu Köln, Number 17-388, date 26 October 2017). Written informed consent will be given by all subjects before study inclusion and randomization. The pseudonymized data management is guaranteed by the data trustee. The study is performed in accordance with German national laws and guidelines, Good Clinical Practice, and the Declaration of Helsinki. The study is registered at ClinicalTrials.gov (trial number NCT03445429).

#### Dissemination policy

Trial results will be published by the authors of this protocol in scientific journals and on ClinicalTrials.gov.

#### 3D- and 4K-display system

A commercially available passive polarizing 3D laparoscopic system consisting of the Einstein Vision® 2.0, 30° camera, 10 mm, 3D full high-definition 32“monitor, Aesculap AG, Tuttlingen, Germany is used. Also, a commercially available 4K system, the Visera 4K UltraHighDefinition, 30° camera, 10 mm, 4K big screen 55” monitor, Olympus Medical system, Olympus Europa SE & Co. KG, Hamburg, Germany is used. The position of the complete laparoscopic training parkour, the camera position in the laparoscopic training system, and the distance from study subject to the screens are standardized. All positions are marked with signs on the training system or on the floor in the operating theater.

#### Laparoscopic training parkour

**Fig. 2:**
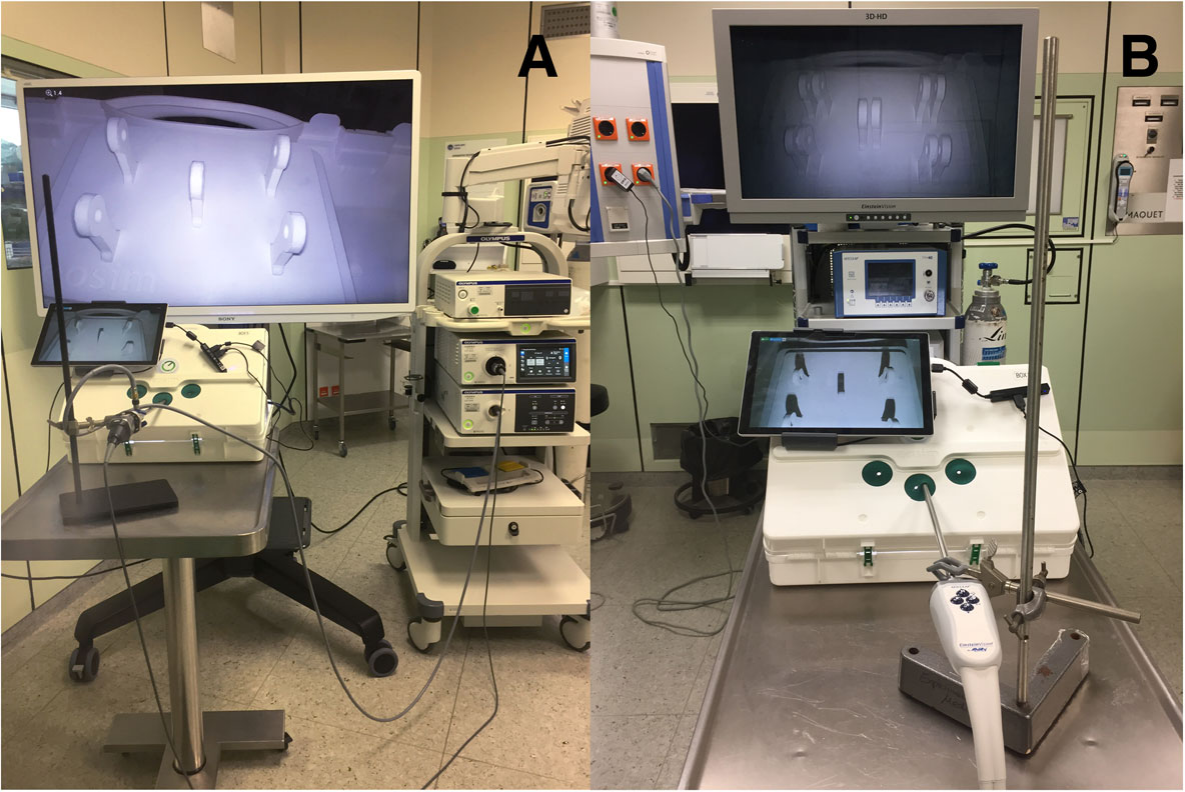
Set-Up - Laparoscopic training parkour. Laparoscopic training simulator in combination with the 4K (**a**) and the 3D Display system (**b**)

**Fig. 3:**
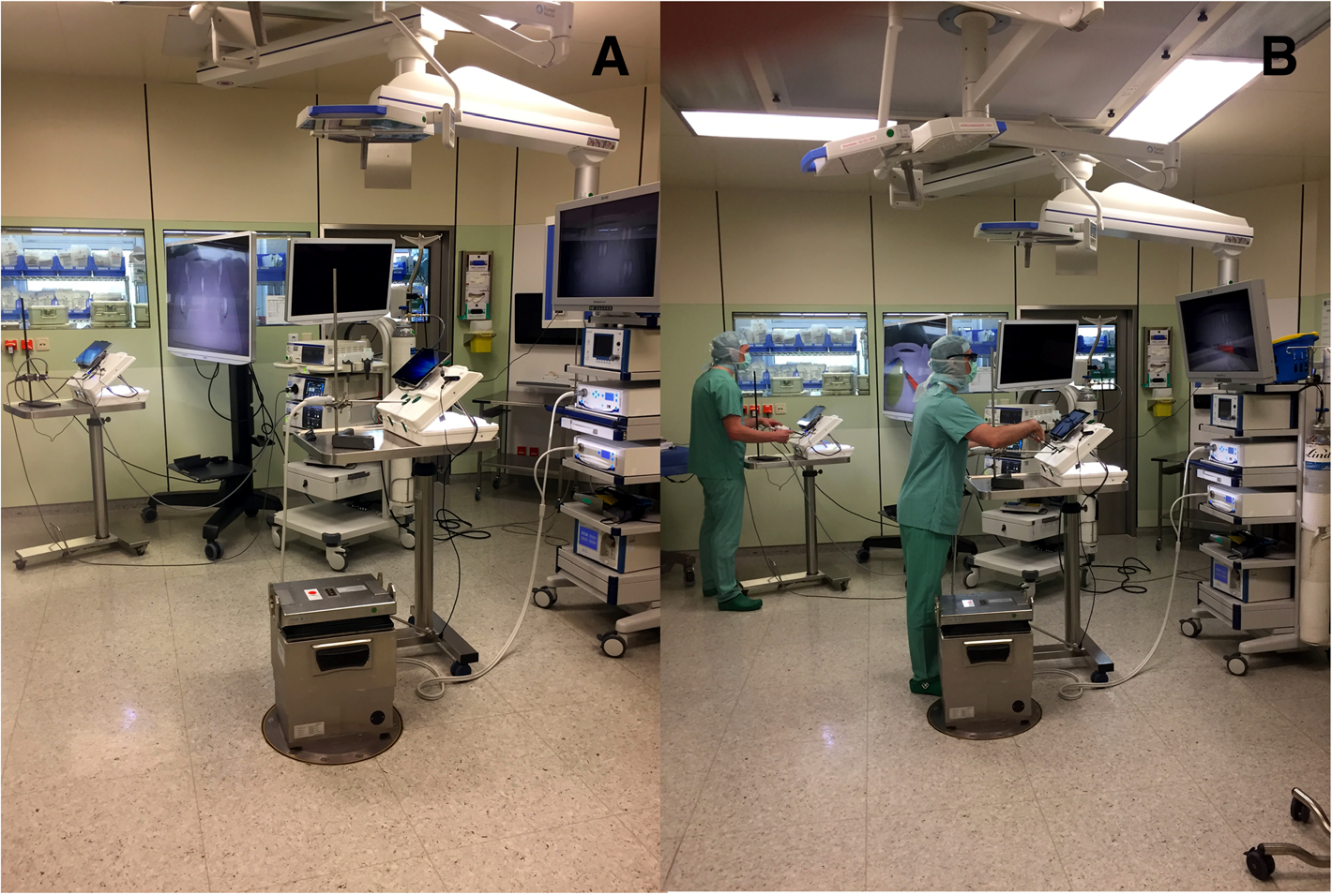
Set-up of the laparoscopic training parkour in the operation theater. Laparoscopic training parkour in the operation theater (**a**, **b**). When using the passive polarizing 3D display system the participating surgeons have to wear special glasses (**b**)

**Fig. 4:**
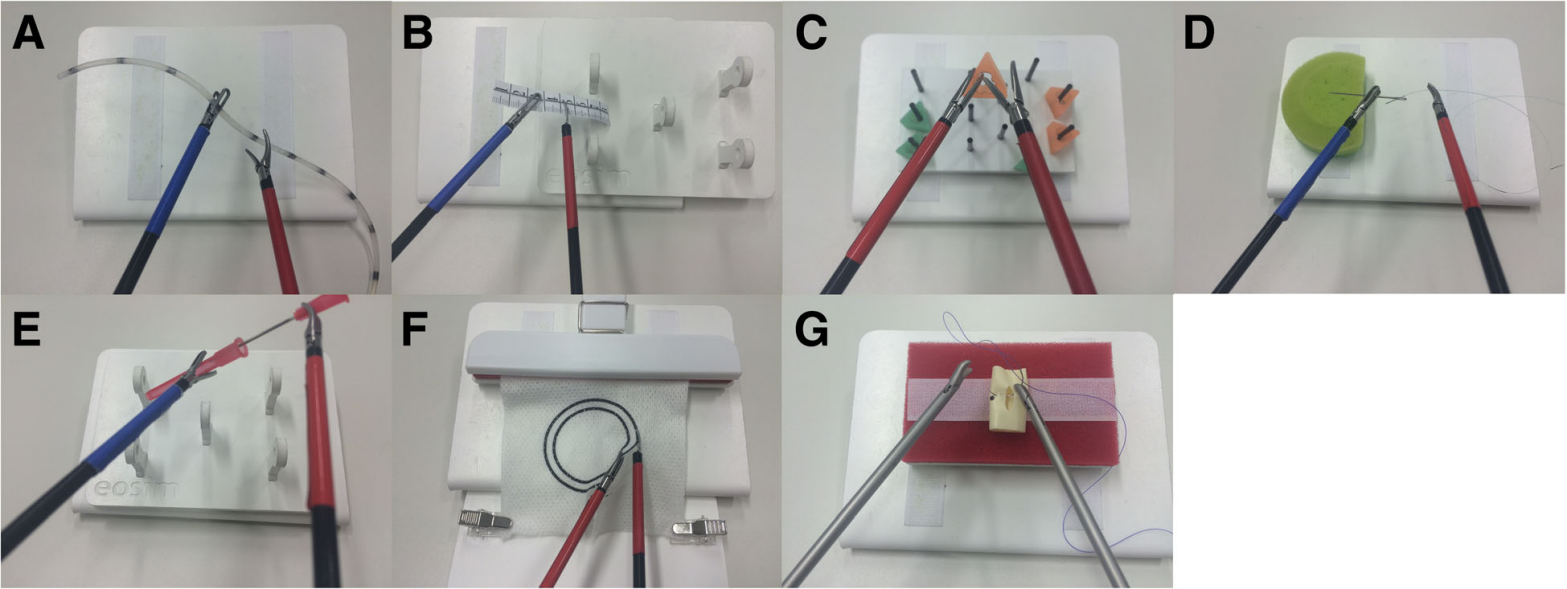
Tasks of the laparoscopic training parkour. Rope pass (**a**), paper cut (**b**), pegboard transfer (**c**), needle threading (**d**), needle recapping (**e**), circle cutting (**f**), and knot tying (**g**)

The laparoscopic training parkour consists of the training simulator (eoSim, eoSurgical Ltd., Edinburgh, UK) wherein the tasks are performed. eoSIM is compatible with the Fundamentals of Laparoscopic Surgery (FLS) trainer. Construct validity for the system was shown previously [13]. Integrated in the training simulator is a video camera system connected to a standard tablet computer. It documents the tasks for evaluation in the 2D video standard. The training simulator is connected to the 3D or 4K display system via the main camera and monitor. The complete setup of the parkour is shown in Figs. 2 and 3. To minimize the potential bias of two simultaneous participants at a training parkour described by Kowalewski et al. [14], participants start in a “time delayed” manner. The display systems are placed 1.5 m away from each other. The working direction is turned by 45°, so that the participants are looking in different directions. Additionally, there are always two investigators in the operation theater, who observe the participants and prevent “copying”. Seven different tasks (novices 5) with increasing difficulty are performed by the subjects. Each task is performed three times in a row. Then the next task follows. The tasks are called “rope pass”, “paper cut”, “pegboard transfer”, “needle threading”, “needle recapping”, “circle cutting” and “knot tying” (Fig. 4). Table 2 briefly describes the tasks.

**Table 2:** Description of the task performed during the laparoscopic training parkour - 3D- vs. 4K-displaysystem

	Tools	Description of task	Special mistakes
Task			
Rope pass	30 cm long silicon tube, marked every 3 cm, mark width 3 mm	Rope should be given from one hand to the other, only grasping at the marks	grasping the non-marked area
Paper cut	8 cm long paper ruler, mark width 1 mm	Make a 0,5 cm long cut every 1 cm on the ruler	Cut through the paper or in non-marked area
Pegboard transfer	Pegboard with 8 triangles, each placed on bars	mid-air transfer of triangles from left to right, back and forth	placing the triangles wrong
Needle threading	Needle on a pin cushion, surgical thread	Needle should be hold in midair, thread should be laced through the eye of the needle	Thread slipped from the eye of the needle
Needle recapping	18-gauge cannula and cap	Cannula should be recapped in mid-air	Cannula touches cap on the outside
Circle cutting^a^	Gauze with a 5 cm diameter two-lined circle, distance between the lines 5 mm	Circle should be cut out of the gauze between the lines	cut out of the marked area
Knot tying^a^	Surgical thread, vessel dummy with opening	one stitch suture with intracorporal knot should be performed	Slipping of thread

^a^not performed by the novice group

Before starting, the participants are shown a video on how to perform the laparoscopic training parkour tasks. They are also given a handout describing all the tasks. After that, no further explanations by the investigators will be given. Color-coded standard laparoscopic instruments are used (grasper, Overholt clamp, scissors, needle holders). Each task starts with the first touch of the used tool and ends with drop-down to the bottom of the laparoscopic trainer box. Performance time is measured between these positions. Mistakes are documented as any deviance from perfect performance. There are general and special mistakes for each task. General mistakes are dropping of the main objects of the tasks (e.g. the rope, paper, needle or thread on the floor of the box trainer), regrasping of the used objects and touching parts of the box trainer. A special mistake e.g. in “rope passing” is grasping the non-marked area, for “paper cut” a too long cut through the paper and for “knot tying” a slipping of the prepared loop. In addition the task will be rated according to the Global Operative Assessment of Laparoscopic Skills (GOALS) [15].

## Discussion

More and more complex surgical procedures are performed in minimally invasive/laparoscopic technique. Optimal visualization of the surgical field is one of the key aspects in this context: the better a surgeon can see, the more subtle preparation of damageable tissue (e.g. small vessel, liver parenchyma) becomes possible. State-of-the-art display technique supports this progress. This study compares in a randomized controlled setting, the use of 3D vs. 4K display technique and its influence on surgical performance. The hypothesis is that one of both techniques could facilitate minimally invasive surgery. This should result in a shorter operating/performance time and a minimized mistake rate. Finally, this would lead to a better outcome for the patient. Depending on the different factors (e.g. structured teaching programs, talent of the surgeon, kind of operation, equipment), 30–100 procedures could be necessary to adopt a complex minimally invasive operation [3, 4, 9]. It seems possible, that an optimal display system could also optimize this teaching and learning process. It could help novice surgeons to improve faster during their training, especially in times of highly specialized surgical centers, external quality control and bench marking with demanding low complication rates. Experienced surgeons, who have learned over the years to deal with reduced standard 2D vision in MIS, could also benefit from optimal display technique. Reducing the task load by optimal intraoperative vison could help to perform long lasting minimally invasive procedures. In terms of working conditions (e.g. retirement at the age of 67 as a surgeon in Germany), optimized intraoperative vision in MIS seems to become an important aspect in the future. Using an in-vitro setting in the study many aspects could be evaluated easier and less biased compared to a clinical trial. In times of offensive marketing and economical influenced decision making in medicine, the authors hope with this investigator initiated trial to improve evidence in this field of minimally invasive surgery and help to choose optimal equipment for the future operation theater.

### Trial status

This protocol represents the trial protocol version 1.0, first posted on the 7th of February 2018. The recruitment began at the 28th February 2018 and will be completed approximately at the 01th May 2019.

## Additional file

Additional file 1: SPIRIT 2013 checklist: recommended items to address in a clinical trial protocol and related documents. (DOC 122 kb)

## Abbreviations

2D: Two-dimensional
3D: Three-dimensional
4K: 4K resolution
CRF: Case report form
MIS: Minimally invasive surgery
NASA-TLX: NASA Task Load Index
