# Correction to: 3D vs. 4 K Display System - Influence of “State-of-the-art”-Display Technique On Surgical Performance (IDOSP-Study) in minimally invasive surgery: protocol for a randomized cross-over trial

**DOI:** 10.1186/s13063-020-4173-y

**Published:** 2020-03-12

**Authors:** Roger Wahba, Rabi Raj Datta, Andrea Hedergott, Jana Bußhoff, Thomas Bruns, Robert Kleinert, Georg Dieplinger, Hans Fuchs, Caroline Gietzelt, Desdemona Möller, Martin Hellmich, Christiane J. Bruns, Dirk L. Stippel

**Affiliations:** 1 Department of General, Visceral and Cancer Surgery, University Hospital of Cologne, University of Cologne, Kerpener Straße 62, 50937 Cologne, Germany; 2 Department of Ophthalmology, University Hospital of Cologne, University of Cologne, Cologne, Germany; 3 Faculty of Management, Economics and Social Sciences, Department of Business Administration and Health Care Management, University of Cologne, Cologne, Germany; 4 Institute of Medical Statistics and Computational Biology, Faculty of Medicine and University Hospital of Cologne, University of Cologne, Cologne, Germany

**Correction to: Trials (2019) 20:299.**

**https://doi.org/10.1186/s13063-019-3330-7**

After publication of our article [1] the authors have notified us that one of the names has been incorrectly spelled.

- Original name spelling:
  Caroline Giezelt.
- Correct name spelling:
  Caroline Gietzelt.
